# Circ-100290 Positively Regulates Angiogenesis Induced by Conditioned Medium of Human Amnion-Derived Mesenchymal Stem Cells Through miR-449a/eNOS and miR-449a/VEGFA Axes

**DOI:** 10.7150/ijbs.39895

**Published:** 2020-05-18

**Authors:** Zichun Tang, Xiaoyue Wu, Liping Hu, Yijing Xiao, Junling Tan, Siyu Zuo, Ming Shen, Xiaoqin Yuan

**Affiliations:** 1 Department of Stomatology, Tongling People's Hospital, Tongling, China; 2 Jiangsu Key Laboratory of Oral Diseases, Nanjing Medical University, Nanjing, China; 3 Department of Dental Implant, Affiliated Hospital of Stomatology, Nanjing Medical University, Nanjing, China; 4 Nanjing Medical University, Department of Anatomy, Histology and Embryology, Nanjing, China; Zichun Tang and Xiaoyue Wu contributed equally to this work.

**Keywords:** circular RNA 100290, amnion-derived mesenchymal stem cells, angiogenesis, human umbilical vein endothelial cells, eNOS, VEGFA

## Abstract

The powerful pro-angiogenic capacity of human amnion-derived mesenchymal stem cells (hAMSCs) could be a valuable therapeutic angiogenesis strategy for bone regeneration. However, the molecular mechanisms underlying this process remain largely unknown. Herein, we report upregulated expression of circular RNA 100290 (circ-100290) and an enhanced angiogenic phenotype of human umbilical vein endothelial cells (HUVECs) incubated with conditioned medium from hAMSCs (hAMSC-CM), whereas downregulation of circ-100290 reversed the pro-angiogenic capacity of HUVECs induced by hAMSC-CM. Circ-100290/microRNA 449a (miR-449a)/endothelial nitric oxide synthase (eNOS) and circ-100290/miR-449a/vascular endothelial growth factor A (VEGFA) axes were predicted by a bioinformatics method and subsequently verified by luciferase reporter assays *in vitro*. Gain- or loss-of-function assays were then performed using small interfering RNAs (siRNAs) targeting circ-100290, or a plasmid overexpressing circ-100290. As expected, downregulation of circ-100290 in HUVECs led to weakened tube formation and migration of HUVECs following hAMSC-CM treatment, along with decreased expression of eNOS and VEGFA. In contrast, upregulation of circ-100290 led to enhanced tube formation and migration of HUVECs following hAMSC-CM treatment, along with increased expression of eNOS and VEGFA. Furthermore, a miR-449a inhibitor could largely rescue the effect of circ-100290 silencing on HUVECs, whereas a miR-449a mimic could significantly rescue the effect of overexpressing circ-100290 on HUVECs. Functional assays using eNOS or VEGF receptor inhibitors indicated eNOS and VEGFA may be important targets of miR-449a. Finally, a Matrigel plug assay revealed weakened angiogenesis when circ-100290 was silenced in HUVECs, but enhanced angiogenesis when circ-100290 was overexpressed *in vivo*. Our results suggest that circ-100290 might function via miR-449a/eNOS and miR-449a/VEGFA axes in the pro-angiogenic role of hAMSC-CM on HUVECs.

## Introduction

Bone regeneration is a complex process involving highly orchestrated events; among them, angiogenesis is of great importance 1. Blood vessels mediate the transport of essential nutrients and oxygen, as well as the removal of waste products during the process of bone regeneration. To this end, several recent studies indicated a relationship between osteogenesis and angiogenesis during bone regeneration 2, 3. In addition, researchers have found that stem cells show potentially great pro-angiogenesis properties during bone regeneration 4. A recent study reported that mesenchymal stem cells (MSCs) can promote angiogenesis of endothelial cells in a paracrine manner both *in vivo* and *in vitro* 5. Our previous study showed that human amnion-derived mesenchymal stem cells (hAMSCs) exhibit a powerful ability to enhance tube formation of human umbilical vein endothelial cells (HUVECs) 6. Moreover, hAMSCs exhibit reduced anti-inflammatory properties and are a more abundant resource of isolated primary cells compared with MSCs 7.

Circular RNAs (circRNAs) are a special class of endogenous non-coding RNAs formed by back splicing with a loop structure outlook 8. Over the past few decades, circRNAs were considered to be a byproduct of transcription. However, with the recent development of next-generation sequencing and bioinformatics, an increasing number of investigations have demonstrated that circRNAs are widely expressed and many are involved in processes of development and disease 9, 10. Initially, circ-CDR1as and circ-Sry were found to act as competing endogenous RNAs (ceRNAs) to bind miRNAs, thereby regulating relevant mRNAs 11, 12. Subsequently, circRNAs were found to be involved in regulation of transcription and alternative splicing, and can even encode proteins 13-16. However, whether circRNAs participate in angiogenesis and the molecular mechanisms involved remain largely unknown.

Recently, a few studies reported that circRNAs may play a role in pathologic angiogenesis. Moreover, a recent investigation suggested that has_circ_0054633 plays an important role in protecting HUVECs from high-glucose damage by sponging miR-218 and, hence, upregulating expression of ROBO1/HO-1 17. Another study indicated the involvement of circ-SHKBP1 in angiogenesis of glioma-exposed endothelial cells via miR-544a/FOXP1 and miR-379/FOXP2 pathways 18. These intriguing studies shed light on the potential molecular mechanisms by which circRNAs participate in pathologic angiogenesis. However, whether circRNAs regulate physiologic or therapeutic angiogenesis remains unclear.

In this study, we found that circ-100290 (has_circ_0013339), which was recently reported to be important in cancer 19, 20, was highly expressed in HUVECs when co-cultured with conditioned medium (CM) of hAMSCs (hAMSC-CM). Furthermore, we demonstrated that circ-100290 may promote the angiogenic capacity of HUVECs by binding miR-449a and subsequently enhancing the expression of endothelial nitric oxide synthase (eNOS) and vascular endothelial growth factor A (VEGFA) induced by hAMSC-CM.

## Materials and Methods

### Ethics statement

Study protocols, which were strictly adhered to, were approved by the Ethics Committee of the School of Stomatology, Nanjing Medical University, China (No. PJ2013-037-001). Methodologies employed in this study were in accordance with the Declaration of Helsinki, and informed consent was collected from each donor recruited in this investigation.

### Cell culture

Identification and isolation of hAMSCs were conducted according to a previously described method 13. Briefly, culture of hAMSCs was performed in α Minimum Essential Medium (α-MEM; Gibco, Grand Island NY, USA) containing 10% fetal bovine serum (FBS, Gibco), 100 U/L penicillin, and 100 mg/L streptomycin. hAMSCs at passages 3-5 were employed in all experiments. HUVECs purchased from China Infrastructure of Cell Line Resources (Beijing, China) were cultured in Dulbecco's Modified Eagle Medium (DMEM, Gibco) containing 10% FBS, 100 U/L penicillin, and 100 mg/L streptomycin. Three donors were enrolled in this study for isolation of hAMSCs.

L-NAME (Beyotime Biotechnology, Shanghai, China) was used as a specific inhibitor of eNOS in HUVECs by pre-treating with 50 nM overnight. AMG706 (Beyotime Biotechnology) was employed as an inhibitor of VEGFRs in HUVECs by pre-treating with 10 nM for 2 hours.

### Collection of hAMSC-CM

After reaching approximately 70%-80% confluency in 15-cm plates, hAMSCs were washed three times with phosphate-buffered saline (PBS). Next, hAMSCs were incubated with 15 mL of serum-free α-MEM containing penicillin and streptomycin, and 15 mL of serum-free DMEM containing penicillin and streptomycin. After 24 h, the medium was collected, centrifuged at 1500 × g for 5 mins, centrifuged at 3000 × g for 3 mins at 4°C, filtered with a 0.45-μm filter (Merck Millipore, Darmstadt, Germany), and ultimately stored at -80°C as hAMSC-CM. During CM collection, hAMSCs from the three donors were separately collected as three repeats for the experiments in this study. In addition, harvesting of hAMSC-CM was performed in triplicate, unless otherwise stated. For all experiments, 80% v/v of hAMSC-CM in fresh medium was used because our previous results showed that this ratio ideally promotes the viability of HUVECs. Identical serum-free medium not incubated with hAMSCs was used as a control medium.

### Tube formation assay

Ninety-six-well plates coated with Matrigel Growth Factor Reduced (BD Biosciences, Franklin Lakes, NJ, USA) were prepared and seeded with HUVECs at a density of 3 × 10^3^ cells/well. After culture for 4-6 hours with hAMSC-CM or control medium (both containing 1% FBS), five randomly selected fields of tube formation were imaged using an optical microscope (Zeiss, Melville, NY, USA). Quantitative analysis of total tube length in each well was conducted with ImageJ software (National Institutes of Health, Bethesda, MD, USA; http://imagej.nih.gov/ij/).

### Wound healing assay and Transwell migration assay

For wound healing assays, HUVECs in logarithmic growth phase were seeded to form confluent monolayers in six-well plates. A straight line was scraped with a pipette tip to form a gap across the cell monolayer. Cell debris left near the gaps was removed by washing with PBS three times. hAMSC-CM or control medium was then added with 2% FBS. Microscopic images were obtained at 0 and 16 h. For each image, the distance of gaps was measured using ImageJ software. For each group, five random locations of each gap were measured.

The migration of HUVECs grown in logarithmic phase *in vitro* was measured using 24-well Transwell inserts with a 6.5-mm diameter and 8- nm pore (Corning, NY, USA). A total of 1.5 × 10^4^ cells/well were suspended in 200 μL of control medium without FBS, and loaded into the upper chambers. Next, 600 μL of hAMSC-CM or control medium (both containing 10% FBS) was added into the lower chambers. After 12-h incubation, migrated cells that had attached to the lower surface of the filter were fixed with 4% paraformaldehyde and then stained with crystal violet. Cells were counted and photographed in five random fields under a digital microscope (Carl Zeiss Microscopy GmbH, Oberkochen, Germany). Wound healing and Transwell migration assays were replicated three times independently.

### RNA interference, miRNA inhibitors, and transfection

Circ-100290 specialized small interfering RNAs (siRNAs), miR-449a inhibitor, and miR-449a mimic, along with their respective si-NC, miR-449a inhibitor-NC, and miR-449a mimic-NC, were purchased from RiboBio (Guangzhou, China). Plasmids for overexpressing circ-100290 (oe-circ) and its respective negative control (oe-NC) were purchased from Genechem (Shanghai, China). Transfections were performed with Lipofectamine 2000 transfection reagent (Invitrogen, Waltham, MA, USA) according to the manufacturer's protocol. After 48-h transfection, circ-ABCB10, eNOS*,* and VEGFA levels were assessed by qPCR. Sequences of qPCR primers are shown in Table S1.

### RNA immunoprecipitation (RIP)

RIP was conducted employing an EZ-Magna RIP Kit (Merck Millipore) following the manufacturer's instructions. Briefly, HEK293T cells were transfected with miR-449a mimic or miR-449a mimic-NC and were lysed with RIP lysis buffer. The lysates were incubated with magnetic beads conjugated with anti-Ago2 (Abcam) or anti-IgG rotating overnight at 4°C. Then the proteins in the immunoprecipitants were removed after digested with protein K. Finally, the purified RNA was detected by RT-qPCR to measure the level of miR-449a and circ-100290.

### Luciferase reporter assay

Circ-100290 sequences with predicted miR-449a binding sites and respective mutated sequences were cloned into the pmiR-RB-Report plasmid (Genechem) after amplification by PCR, and named circ-WT and circ-MUT, respectively. The same procedure was performed for eNOS and VEGFA. Plasmids were then co-transfected into HEK293T cells or HUVECs with miR-449a mimic or miR-449a mimic-NC, as indicated. Dual-luciferase assays (Promega, Madison, WI, USA) were then performed according to the manufacturer's instructions. Plasmids used in this study and gene sequences are shown in Table S1.

### Western blot analysis

Culture medium was discarded when cells reached 80% confluence. Subsequently, cells were washed with pre-chilled 1× PBS three times and 300 μL of RIPA buffer was added for cellular protein extraction. After incubation for 30 mins on ice, lysed cells were moved into a 1.5-mL centrifuge tube and centrifuged at 4°C for 15 mins at 12,000 rpm. A bicinchoninic acid assay was performed for protein quantification. Protein samples were fractionated by sodium dodecyl sulfate-polyacrylamide gel electrophoresis and electrotransferred to polyvinylidene fluoride membranes (Merck Millipore). Next, membranes were incubated with a primary antibody against VEGFA (Abcam, Cambridge, UK; Cat. No. ab171828; 1:200) at room temperature for 1 h or GAPDH (Abcam; Cat. No. ab8245; 1:1000), eNOS [Cell Signaling Technology (CST), Danvers, MA, USA; Cat. No. 9586; 1:500], ERK1/2 (CST; Cat. No. 4695; 1:1000), or p-ERK1/2 (CST; Cat. No. 9586; 1:1000) at 4°C overnight. Subsequently, membranes were washed with 0.1% Tween-20 in Tris-buffered saline, and then incubated with a secondary antibody conjugated to horseradish peroxidase at room temperature for 1 h. Finally, enhanced chemiluminescence detection was performed.

### Quantitative real-time PCR

Quantity and quality of total RNA was measured using Nanodrop 2000 spectrophotometry (Thermo Fisher Scientific, Waltham, MA, USA) after extraction with TRIzol reagent (Invitrogen). A Prime Script RT Master Mix (Takara Bio, Shiga, Japan) was used to reverse-transcribe 1 µg of total RNA to cDNA, while a TaqMan^®^ MicroRNA Reverse Transcription kit (Applied Biosystems, Foster City, CA, USA) was used to reverse-transcribe miRNA with specific primers. Expression levels of respective RNAs were detected using SYBR Green Master Mix (Takara Bio) on an ABI 7500 system (Thermo Fisher Scientific) according to the manufacturer's instructions. Relative fold-changes of RNA expression were then calculated with the ΔΔCt method using GAPDH or U6 as the internal standard. RT-qPCR analysis was performed three times independently.

For extraction of nuclear-cytoplasmic separated RNA, a PARIS™ Kit (Thermo Fisher Scientific) was used according to the manufacturer's protocol. U6 was used as the internal standard for nuclear RNA. Primer sequences used in this study are listed in Table S1.

### Measurement of endothelial NO production

Production of NO in HUVECs of each group was detected employing the NO-specific fluorescent dye 3-amino-4-aminomethyl-2',7'-difluorescein, diacetate (DAF-FM DA) (Beyotime Biotechnology). Briefly, each group of live HUVECs were incubated with 5 nM DAF-FM DA for 40 mins at 37°C, and then washed three times with PBS. Dynamic changes of fluorescence intensity were measured in cells using a SPECTRONIC™ 200 spectrophotometer (Thermo Fisher Scientific) at an excitation wavelength of 495 nm. Images obtained were analyzed using ImageJ Software Version 1.40. Fluorescence intensity of DAF-FM was first normalized to the fluorescence area, and then normalized to that of the respective control group.

### Statistical analysis

All data in this study were obtained and analyzed from three independent repeats in a double-blind manner. All image analysis was conducted by a blinded observer. All data are expressed as mean ± standard error unless otherwise stated, and the results were analyzed with SPSS 20.0 software (IBM, Armonk, NY, USA). Statistical comparisons were made using one-way repeated-measures ANOVA or two-tailed Student's *t*-test with post-hoc tests. Differences were considered to be statistically significant when *P* < 0.05.

## Results

### Downregulation of circ-100290 inhibited the pro-angiogenic role of hAMSC-CM *in vitro* and *in vivo*

**Figure 1:**
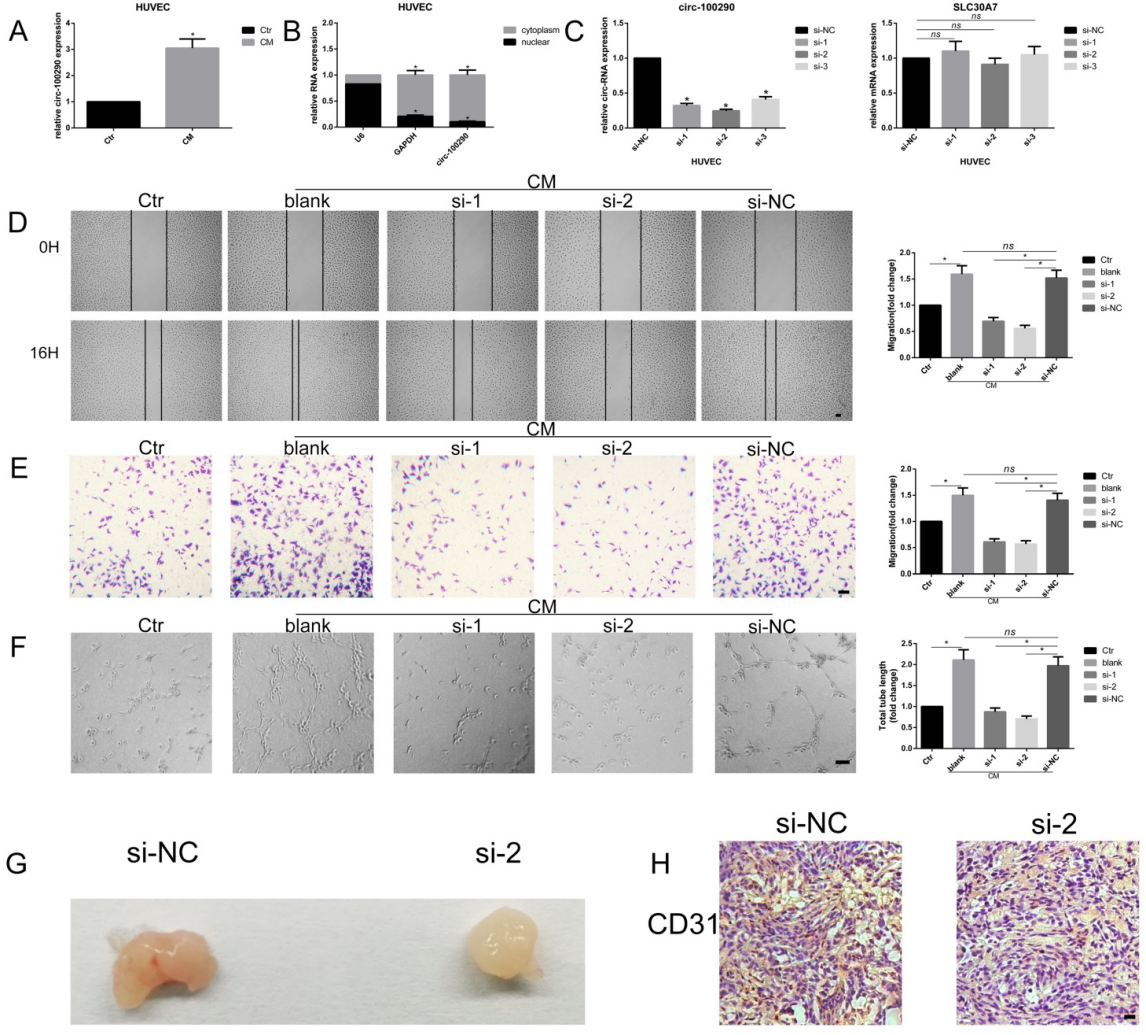
** Downregulation of circ-100290 inhibits the pro-angiogenic role of hAMSC-CM *in vitro* and *in vivo*.** (A) Fold change of circ-100290 in HUVECs treated with hAMSC-CM were measured by qPCR. *P<0.05 (B) Distribution of circ-100290 in HUVECs measured by qPCR via nuclear-cytoplasmic separating method. *P<0.05 (C) Three specific siRNAs targeting circ-100290 were transfected into HUVECs, and the efficiency and the relative expression of circ-100290 and SLC30A7 (linear form of circ-100290) were measured by qPCR. *P<0.05 (D, E) Scratch test and transwell assays showed the effect of downregulation of circ-100290 on HUVECs' migration induced by hAMSC-CM. *P<0.05. Scale bar: 100μm. (F) Tube formation assay showed the effect of circ-100290 downregulation on HUVECs angiogenesis induced by hAMSC-CM. *P<0.05. Scale bar: 100μm. (G, H) Matrigel plug assay and immunohistochemistry for CD31 was conducted to evaluate the angiogenic capacity effected by si-2. *P<0.05. Scale bar: 100μm. ALL data were analyzed using one-way repeated-measures ANOVA and two-tailed Student *t*-test with post-hoc tests, error bar represented SEM. Ctr: control; NC: negative control.

Our previous study confirmed that hAMSC-CM effectively enhanced tube formation and migration of HUVECs (data not shown). We found that expression of circ-100290 was dramatically upregulated in HUVECs treated with hAMSC-CM. As such, we examined whether circ-100290 plays an important role in the pro-angiogenesis effect induced by hAMSC-CM (Fig. 1A). Recent studies showed that circ-100290 mainly localizes in cytoplasm, whereby it has important functions in cancer cells 20. Our qPCR also verified that circ-100290 is mainly distributed in the cytoplasm of HUVECs (Fig. 1B). Next, we designed three siRNAs named si-1, si-2, and si-3, that specifically targeted the junction site of circ-100290 in HUVECs. After transfection with these three siRNAs, we measured the efficiency of circ-100290 downregulation in HUVECs by qPCR. Our results showed that si-1 and si-2 were more efficient than si-3 (Fig. 1C). In addition, we detected the level of line form of circ-100290 (the mRNA of *SLC-30A7*). Our results showed no significant change after transfection with any of the three siRNAs in HUVECs (Fig. 1C). Next, we examined whether downregulation of circ-100290 influenced the angiogenic phenotype of HUVECs by performing assays for wound healing, migration, and tube formation. Our results showed that migration and tube formation ability of HUVECs were significantly weakened after downregulation of circ-100290 by transfection with si-1 or si-2 (Fig. 1D-F).

A Matrigel plug assay was performed to further investigate whether downregulation of circ-100290 regulates angiogenesis of HUVECs *in vivo*. HUVECs transfected with si-NC or si-2 were injected into nude mice. General observations revealed that the si-2 group exhibited decreased angiogenesis compared with the si-NC group (Fig. 1G). Furthermore, immunohistochemistry for CD31 was conducted to evaluate the angiogenic capacity of each group. As expected, CD31 staining was lighter in the si-2 group compared with the si-NC group (Fig. 1H).

### Overexpression of circ-100290 enhanced the pro-angiogenic role of hAMSC-CM *in vitro* and *in vivo*

**Figure 2:**
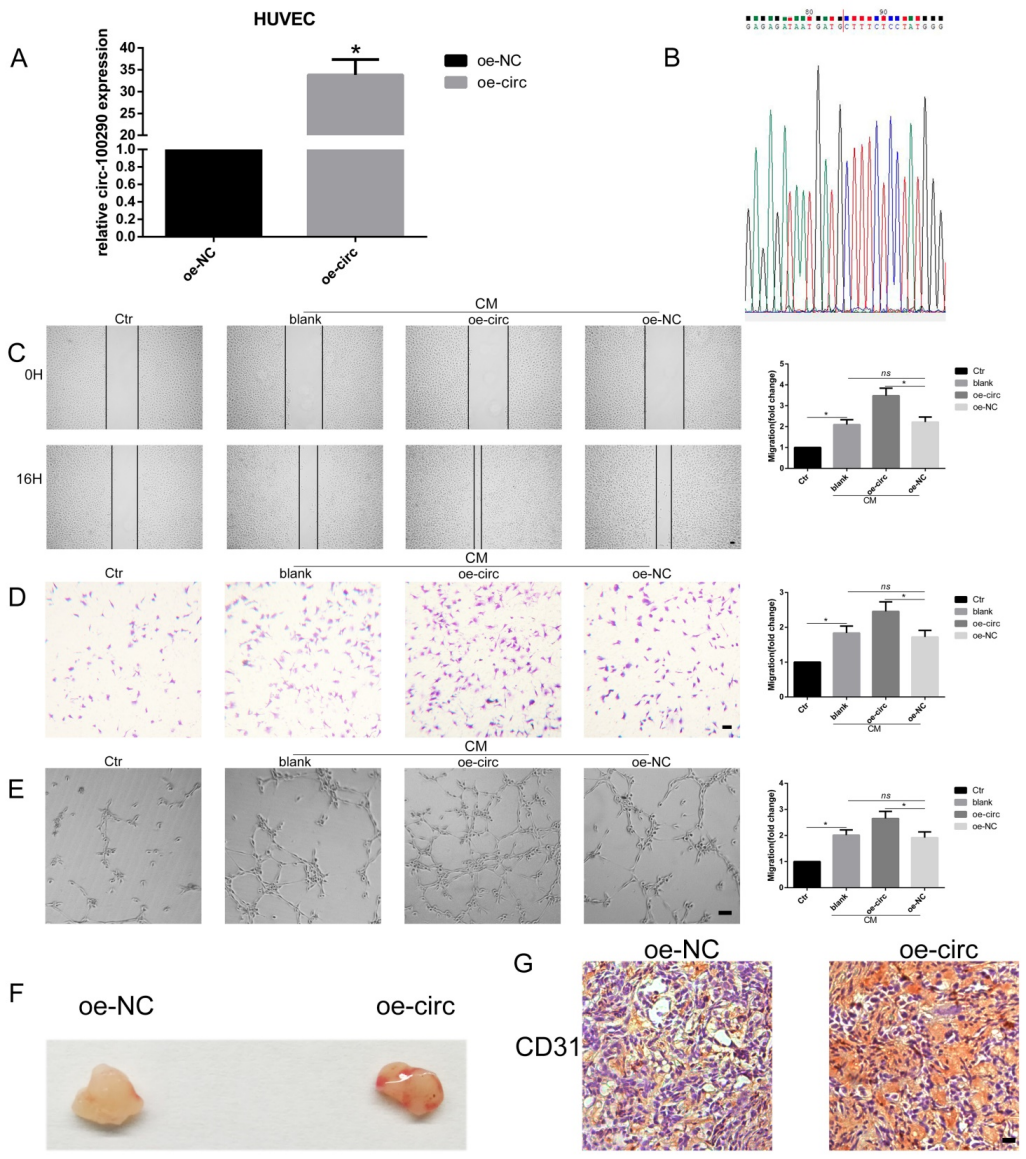
** Overexpression of circ-100290 enhances the pro-angiogenic role of hAMSC-CM *in vitro* and *in vivo*.** (A) Plasmid for overexpression of circ-100290 was transfected into HUVECs, and the efficiency was measured by qPCR. *P<0.05 (B) The sequence of sequencing the product of qPCR contained the special junction sequence of circ-100290. (C, D) Scratch test and transwell assays showed the effect of upregulation of circ-100290 on HUVECs migration induced by hAMSC-CM. *P<0.05. Scale bar: 100μm. (E) Tube formation assay showed the effect of circ-100290 upregulation on HUVECs angiogenesis induced by hAMSC-CM. *P<0.05. Scale bar: 100μm. (G, H) Matrigel plug assay and immunohistochemistry for CD31 was conducted to evaluate the angiogenic capacity effected by overexpressing circ-100290 in HUVECs. *P<0.05. Scale bar: 100μm. ALL data were analyzed using one-way repeated-measures ANOVA and two-tailed Student *t*-test with post-hoc tests, error bar represented SEM. oe: over expression.

After investigating loss-of-function of circ-100290 in HUVECs, we next examined gain-of-function of circ-100290 in HUVECs by designing plasmids for overexpressing circ-100290, named oe-circ and oe-NC. After transfection, we detected circ-100290 levels by qPCR. The results showed approximately 32-fold increased expression of circ-100290 in the oe-circ group compared with the oe-NC group (Fig. 2A). Sequencing of the qPCR product revealed that it contained the special junction sequence of circ-100290 (Fig. 2B). Next, we performed functional assays to elucidate the effect of circ-100290 overexpression on HUVECs. A wound healing assay showed that the oe-circ group exhibited a marked enhancement compared with the oe-NC group (Fig. 2C). Migration and tube formation assays showed similar changes (Fig. 2D and E).

Next, a Matrigel plug assay was conducted to investigate whether overexpression of circ-100290 regulates angiogenesis of HUVECs *in vivo*. HUVECs transfected with oe-NC or oe-circ were injected into nude mice. General observations revealed that the oe-circ group exhibited enhanced angiogenesis compared with the oe-NC group (Fig. 2F). Moreover, immunohistochemistry for CD31 was performed to evaluate the angiogenic capacity of these two groups. As expected, the oe-circ group expressed more CD31 than the oe-NC group (Fig. 2G).

### Circ-100290 regulated the pro-angiogenic role of hAMSC-CM by binding miR-449a, which may target eNOS and VEGFA

**Figure 3:**
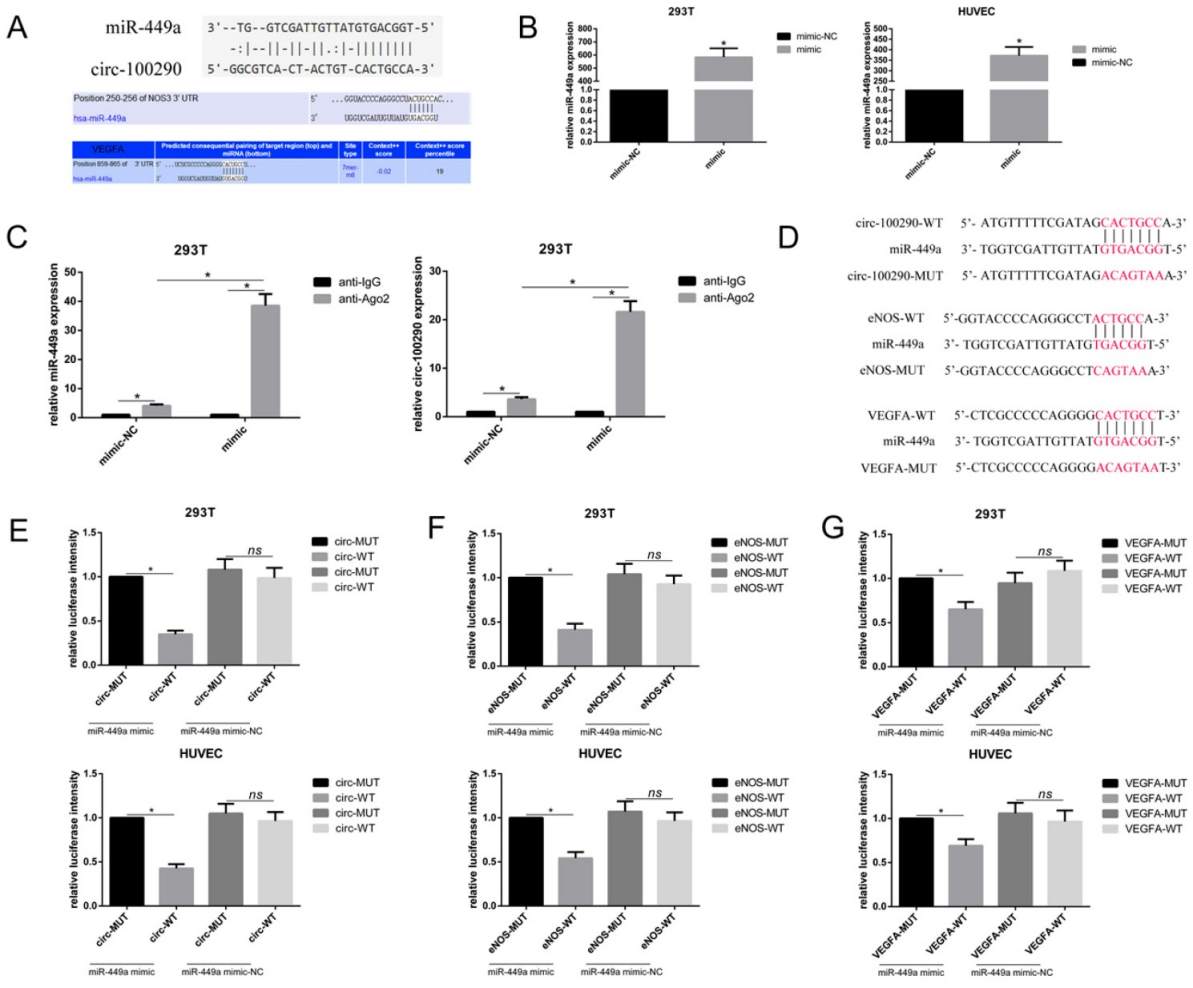
** Circ-100290 regulated the pro-angiogenic role of hAMSC-CM by binding miR-449a which might target eNOS and VEGFA.** (A) Sequences in miR-449a predicted to target circ-100290, eNOS and VEGFA. (B) miR-449a mimic was transfected into 293T cells and HUVECs, the efficiency was measured by qPCR. *P<0.05 (C) RNA immunoprecipitation was performed using anti-IgG or anti-Ago2 in 293T cells transfected with miR-449a mimic or mimic-NC, and the level of miR-449a and circ-100290 in each group was measured by qPCR. *P<0.05 (D) Plasmids containing predicted binding sites or mutant binding sites designed for Dual-luciferase assays. (E, F and G) Dual-luciferase assay. 293T cells or HUVECs were co-transfected with miR-449a mimic or mimic-NC and plasmid with wild-type or mutant circ-100290, eNOS and VEGFA. *P<0.05. ALL data were analyzed using one-way repeated-measures ANOVA and two-tailed Student *t*-test with post-hoc tests, error bar represented SEM. mimic: miR-449a mimic; mimic-NC: miR-449a mimic-NC; WT: wild type; MUT: mutation.

Recent studies showed that circ-100290 may function through binding miRNAs in cancer cells 19, 20. Therefore, we examined whether circ-100290 can regulate angiogenesis of HUVECs through affecting relative miRNAs. Upon analyzing potential circ-100290-binding miRNAs predicted by Targetscan, starBase, and RegRNA, miR-449a drew our attention because it was predicted to potentially target the 3'UTR of both eNOS and VEGFA mRNA (Fig. 3A), which are extremely important during angiogenesis. Indeed, although a recent study found that miR-449a might be involved in prostate cancer-associated angiogenesis 21, whether miR-449a plays a direct role in angiogenesis was unknown. For this purpose, we examined whether circ-100290 regulated eNOS and VEGFA by binding miR-449a, as predicted, by performing RIP and luciferase assays. The efficiency of transfection of miR-449a mimic in HEK293T and HUVEC was detected by qPCR (Fig 3B). As expect, endogenous miR-449a pulldown by anti-Ago2 was significantly increased in HEK293T transfected with miR-449a mimic rather than transfected with miR-449a mimic-NC. And then we detected the level of circ-100290 in each group, the result showed endogenous circ-100290 pulldown by anti-Ago2 was markedly enriched in HEK293T (a widely used standardized tool cell line in the RIP assay) transfected with miR-449a mimic rather than transfected with miR-449a mimic-NC. (Fig. 3C). For Luciferase assays, specific plasmids containing predicted binding sites or mutant binding sites were designed and named circ-WT, circ-MUT, eNOS-WT, eNOS-MUT, VEGFA-WT, and VEGFA-MUT, respectively (Fig. 3D). Luciferase assays were performed in both HEK293T and HUVECs. As expected, relative luciferase intensity was remarkably decreased in the circ-WT + miR-449a mimic group compared with the circ-WT + miR-449a mimic-NC group, while there was no significant difference between circ-MUT + miR-449a mimic and circ-MUT + miR-449a mimic-NC groups (Fig. 3E). Similar results were observed for eNOS and VEGFA (Fig. 3F and G). Hence, we speculated that circ-100290 regulates eNOS and VEGFA by affecting miR-449a.

**Figure 4:**
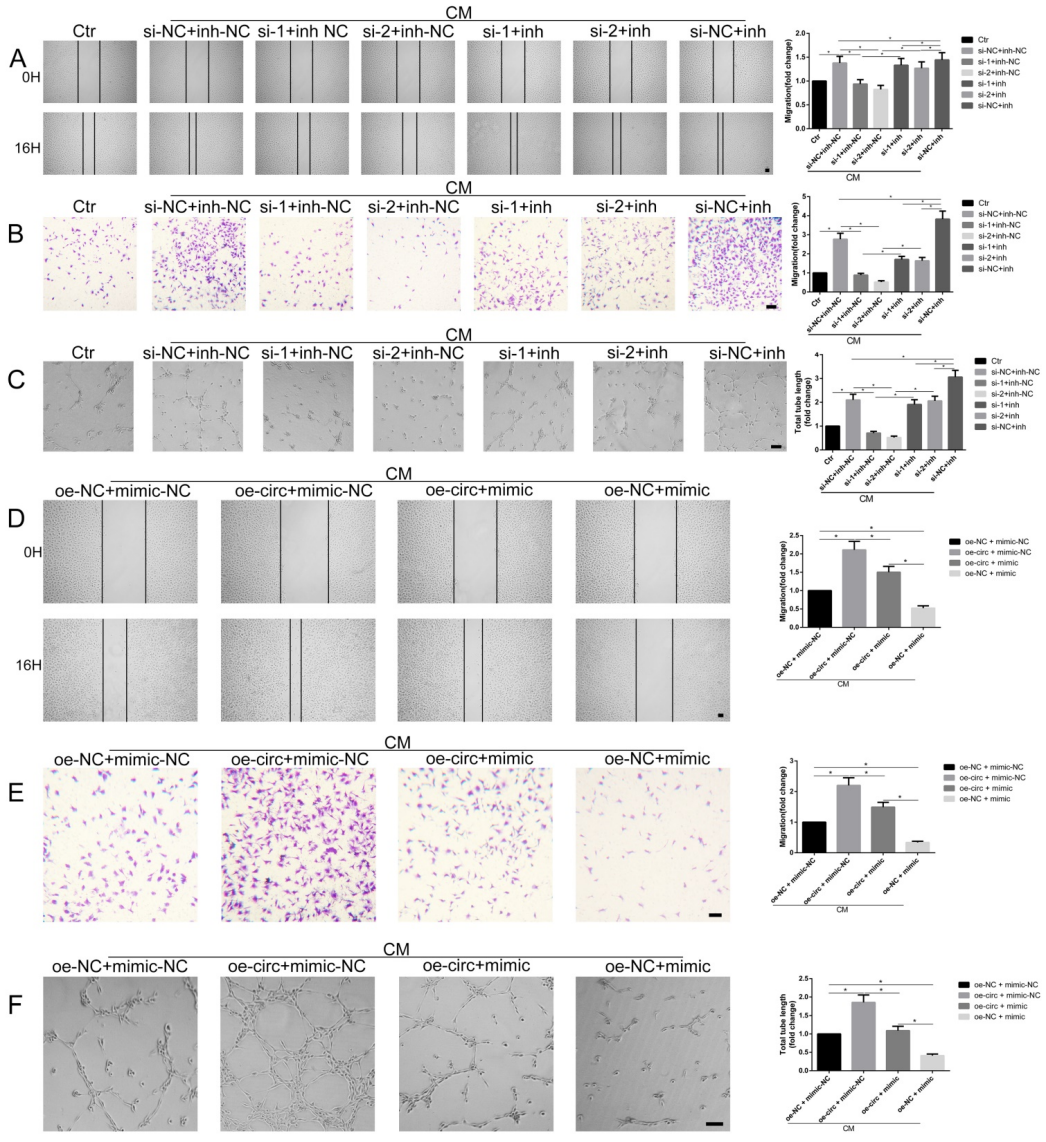
** miR-449a involvement in circ-100290 regulating hAMSC-CM-induced pro-angiogenisis.** (A, B) Scratch test and transwell assays showing the effect of circ-100290 downregulation on hAMSC-CM-induced HUVECs migration was largely rescued by miR-449a inhibitor. *P<0.05. Scale bar: 100μm. (C) Tube formation assay showed the effect of circ-100290 downregulation hAMSC-CM-induced HUVECs angiogenesis was significantly rescued by miR-449a inhibitor. *P<0.05. Scale bar: 100μm. (D, E) Scratch test and transwell assays showing the effect of circ-100290 upregulation on hAMSC-CM-induced HUVECs migration was largely rescued by miR-449a inhibitor. *P<0.05. Scale bar: 100μm. (F) Tube formation assay showed the effect of circ-100290 upregulation hAMSC-CM-induced HUVECs angiogenesis was significantly rescued by miR-449a inhibitor. *P<0.05. Scale bar: 100μm. ALL data were analyzed using one-way repeated-measures ANOVA and two-tailed Student *t*-test with post-hoc tests, error bar represented SEM. inh: inhibitor.

To functionally illustrate the potential involvement of circ-100290 in the process of enhanced angiogenesis by hAMSC-CM via miR-449a, a series of functional assays were conducted in the presence of hAMSC-CM. A scratch test showed that si-1 + miR-449a inh-NC and si-2 + miR-449a inh-NC groups exhibited markedly weakened migration compared with the si-NC + miR-449a inh-NC group. However, this weakened migration was rescued in si-1 + miR-449a inh and si-2 + miR-449a inh groups (Fig. 4A). Similar tendencies were observed in migration and tube formation assays (Fig. 4B and C). In addition, we examined whether miR-449a overexpression could rescue the enhanced angiogenic capacity of HUVECs caused by circ-100290 upregulation. As expected, the scratch test assay showed that migration of the oe-circ + miR-449a mimic group was markedly decreased compared with the oe-circ + miR-449a mimic-NC group; whereas, the oe-NC + miR-449a mimic group showed the weakest migratory ability (Fig. 4D). Similar tendencies were detected in migration and tube formation assays (Fig. 4E and F).

### Circ-100290 regulated hAMSC-CM-induced pro-angiogenesis via the miR-449a/eNOS axis

**Figure 5:**
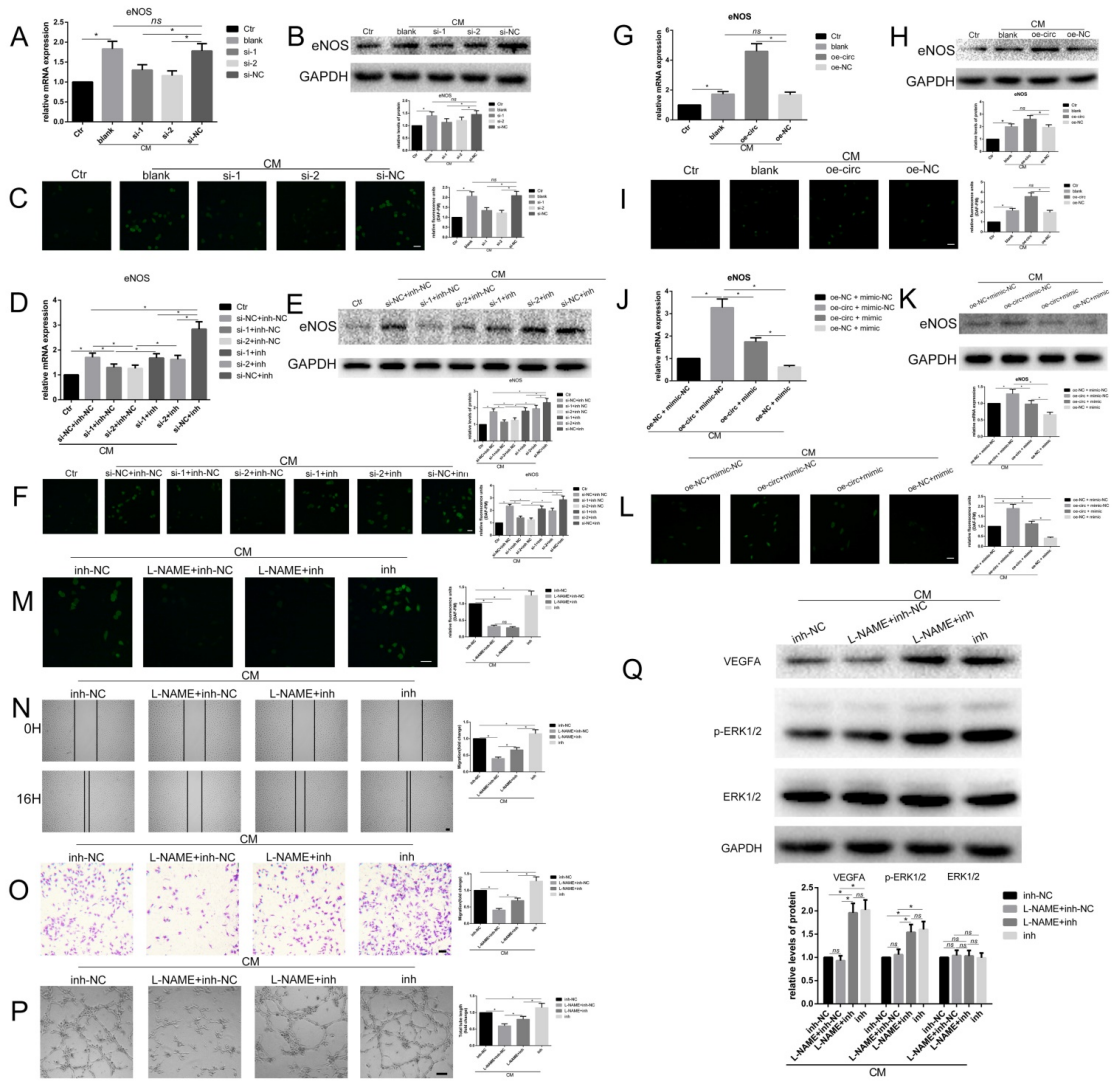
** Circ-100290 regulated hAMSC-CM-induced pro-angiogenisis via miR-449a/eNOS axis.** (A, B) Relative expression change of eNOS effect by circ-100290 downregulation on hAMSC-CM-induced HUVECs in mRNA and protein levels. *P<0.05. (C) Effect of the fluorescence reflecting the enzymatic activity of eNOS caused by downregulation of circ-100290. *P<0.05. Scale bar: 100μm. (D, E) The effect of circ-100290 downregulation on the expression of eNOS in hAMSC-CM-induced HUVECs was largely rescued by miR-449a inhibitor. *P<0.05. (F) Effect of the fluorescence reflecting the enzymatic activity of eNOS caused by downregulation of circ-100290 was largely rescued by miR-449a inhibitor. *P<0.05. Scale bar: 100μm. (G, H) Relative expression change of eNOS effect by circ-100290 upregulation on hAMSC-CM-induced HUVECs in mRNA and protein levels. *P<0.05. (I) Effect of the fluorescence reflecting the enzymatic activity of eNOS caused by upregulation of circ-100290 on hAMSC-CM-induced HUVECs. *P<0.05. Scale bar: 100μm. (J, K) The effect of circ-100290 upregulation on the expression of eNOS in hAMSC-CM-induced HUVECs was largely rescued by miR-449a mimic. *P<0.05. (L) Effect of the fluorescence reflecting the enzymatic activity of eNOS caused by upregulation of circ-100290 was largely rescued by miR-449a mimic. *P<0.05. Scale bar: 100μm. (M) Fluorescent detection showed the enzymatic activity of eNOS was effectively inhibited by L-NAME. *P<0.05. Scale bar: 100μm. (N, O) Scratch test and transwell assays showed L-NAME could only partially abort the effect of miR-449a inhibitor on HUVECs treated with hAMSC-CM. *P<0.05. Scale bar: 100μm. (P) Tube formation assay showed L-NAME could only partially abort the effect of miR-449a inhibitor on HUVECs treated with hAMSC-CM. *P<0.05. Scale bar: 100μm. (Q) Western blotting showed the level of protein of VEGFA and p-ERK1/2 were negatively affected by L-NAME while positively affected by miR-449a inhibitor. *P<0.05. ALL data were analyzed using one-way repeated-measures ANOVA and two-tailed Student *t*-test with post-hoc tests, error bar represented SEM. L-NAME: N^G^-Nitro-L-arginine Methyl Ester, Hydrochloride.

Recently, eNOS has garnered much attention for its role in angiogenesis 22. Thus, we examined whether circ-100290 could regulate the pro-angiogenesis role of hAMSC-CM by the miR-449a/eNOS axis, as predicted, by detecting mRNA and protein levels of eNOS in HUVECs. Our results showed a remarkable increase in HUVECs treated with hAMSC-CM (Fig. 5A and B), as well as a sharp decrease when transfected with si-1 or si-2 compared with transfection of si-NC (Fig. 5A and B). A similar tendency was observed for fluorescence reflecting the enzymatic activity of eNOS (Fig. 5C). Next, we examined whether miR-449a inh could rescue the suppressed expression of eNOS caused by circ-100290 downregulation. Detection of eNOS mRNA and protein levels indicated that si-1 + miR-449a inh-NC and si-2 + miR-449a inh-NC groups exhibited markedly suppressed expression of eNOS compared with the si-NC + miR-449a inh-NC group (Fig. 5D and E). However, suppressed expression of eNOS was rescued in si-1 + miR-449a inh and si-2 + miR-449a inh groups (Fig. 5D and E). A similar tendency was observed for eNOS fluorescence (Fig. 5F).

To illustrate the effect of overexpressing circ-100290 on hAMSC-CM-treated HUVECs, eNOS mRNA and protein levels in HUVECs were detected. As expected, overexpressing circ-100290 in HUVECs upregulated eNOS mRNA and protein levels (Fig. 5G and H). Moreover, fluorescence reflecting the enzymatic activity of eNOS was also upregulated (Fig. 5I). Thus, we examined whether overexpressing miR-449a could rescue the enhanced expression of eNOS in hAMSC-CM-treated HUVECs caused by circ-100290 upregulation. As expected, results of qPCR and western blot revealed markedly decreased eNOS mRNA and protein expression in the oe-circ + miR-449a mimic group compared with the oe-circ + miR-449a mimic-NC group; whereas, the oe-NC + miR-449a mimic group exhibited the lowest eNOS expression (Fig. 5J and K). Fluorescence reflecting the enzymatic activity of eNOS exhibited the same trend (Fig. 5L).

To further investigate whether inhibition of eNOS could abolish the pro-angiogenic effect caused by miR-449a inh on HUVECs treated with hAMSC-CM, we employed L-NAME (an inhibitor of eNOS) to conduct relevant assays. Fluorescent detection showed remarkably decreased fluorescence in L-NAME + miR-449a inh-NC and L-NAME + miR-449a inh groups (Fig. 5M), suggesting the enzymatic activity of eNOS was effectively inhibited. Therefore, we conducted wound healing and migration assays to examine the migration of each group. Unexpectedly, the migratory ability of the L-NAME + miR-449a inh group was significantly weakened compared with the miR-449a inh group, while there was a marked decrease of migration in the L-NAME + miR-449a inh-NC group compared with the L-NAME + miR-449a inh group (Fig. 5N and O). The tube formation assay exhibited the same trend (Fig. 5P). Thus, we assumed that inhibition of eNOS could only partially abolish the effect of miR-449a inh on HUVECs treated with hAMSC-CM. As such, during the promotion of angiogenesis of HUVECs induced by miR-449a inh, there may be other targets besides eNOS regulated by miR-449a. Considering our previous hypothesis that circ-100290 might affect miR-499a to regulate downstream targets eNOS and VEGFA, we performed western blotting. As expected, protein levels of VEGFA and p-ERK1/2 were upregulated in miR-449a inh-treated groups compared with that in L-NAME-treated groups (Fig. 5Q).

### Circ-100290 regulated hAMSC-CM-induced pro-angiogenesis via the miR-449a/VEGFA axis

**Figure 6:**
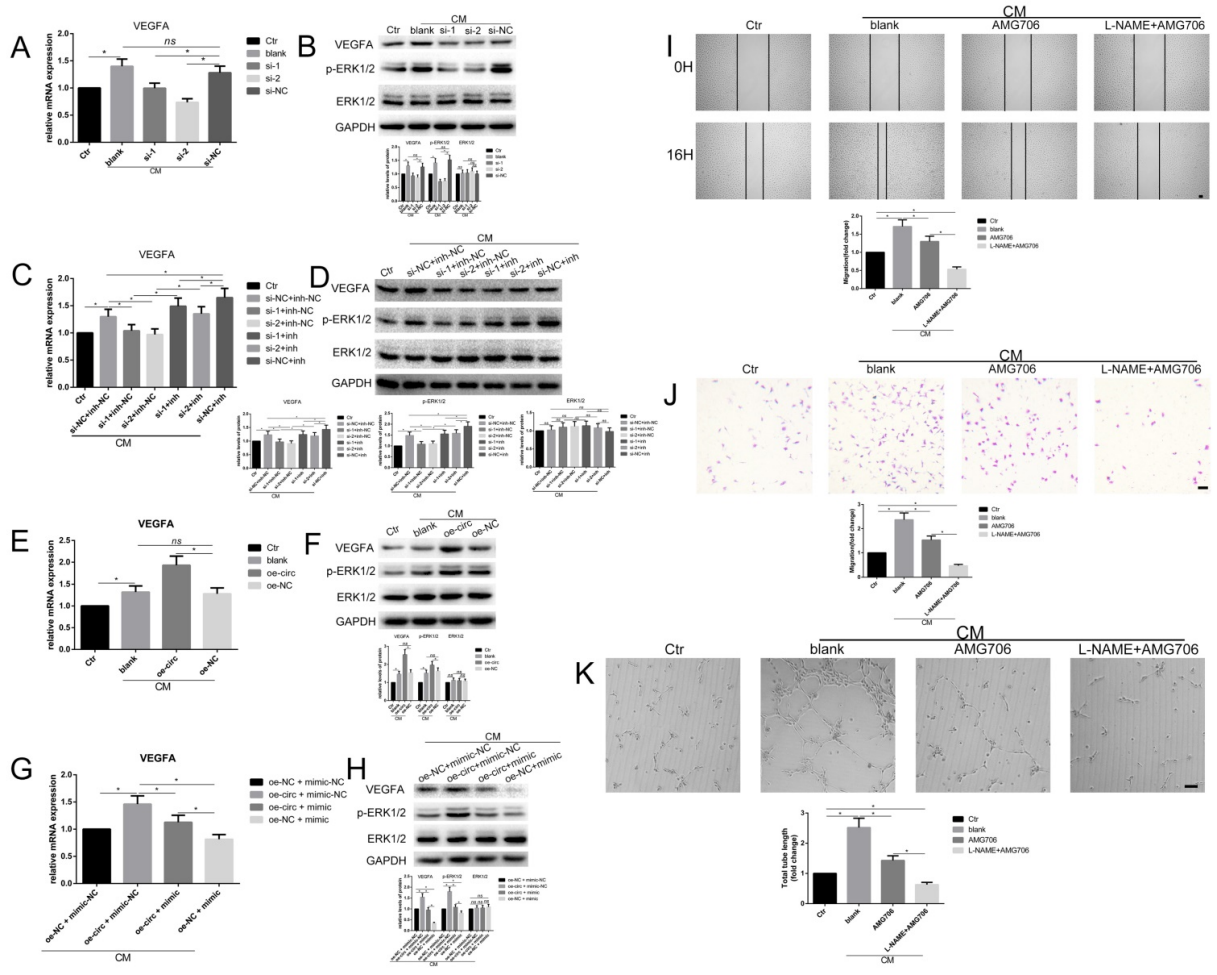
** Circ-100290 regulated hAMSC-CM-induced pro-angiogenisis via miR-449a/VEGFA axis.** (A, B) Relative expression change of VEGFA and p-ERK1/2 effect by circ-100290 downregulation on hAMSC-CM-induced HUVECs in mRNA and protein levels. *P<0.05. (C, D) The effect of circ-100290 downregulation on the expression of VEGFA and p-ERK1/2 in hAMSC-CM-induced HUVECs was largely rescued by miR-449a inhibitor. *P<0.05. (E, F) Relative expression change of VEGFA and p-ERK1/2 effect by circ-100290 upregulation on hAMSC-CM-induced HUVECs in mRNA and protein levels. *P<0.05. (G, H) The effect of circ-100290 upregulation on the expression of VEGFA and p-ERK1/2 in hAMSC-CM-induced HUVECs was largely rescued by miR-449a mimic. *P<0.05. (I, J) Scratch test and transwell assays showed AMG706 could partially abort the enhanced migration in HUVECs induced by hAMSC-CM. *P<0.05. Scale bar: 100μm. (K) Tube formation assay showed AMG706 could partially abort the pro-angiogenic role of hAMSC-CM. *P<0.05. Scale bar: 100μm. ALL data were analyzed using one-way repeated-measures ANOVA and two-tailed Student *t*-test with post-hoc tests, error bar represented SEM. AMG706: Motesanib Diphosphate.

To examine whether circ-100290 can regulate the pro-angiogenesis role of hAMSC-CM via the miR-449a/VEGFA axis, as revealed above, expression of VEGFA was detected by qPCR and western blot. The results showed significantly increased VEGFA expression in HUVECs treated with hAMSC-CM (Fig. 6A and B), as well as a marked decrease upon transfection with si-1 or si-2 compared with si-NC (Fig. 6A and B). Protein levels of p-ERK1/2, which could potentially be activated by VEGFA, exhibited a similar trend (Fig. 6A and B). Furthermore, we determined whether miR-449a inh could rescue the inhibited expression of VEGFA caused by circ-100290 downregulation. As expected, detection of VEGFA mRNA and protein revealed markedly suppressed VEGFA expression in si-1 + miR-449a inh-NC and si-2 + miR-449a inh-NC groups compared with the si-NC + miR-449a inh-NC group (Fig. 6C and D). Thus, suppressed VEGFA expression was rescued in si-1 + miR-449a inh and si-2 + miR-449a inh groups (Fig. 6C and D). A similar trend was observed for expression of p-ERK1/2 (Fig. 6C and D).

Next, we investigated whether overexpressing circ-100290 could regulate the pro-angiogenesis role of hAMSC-CM via the miR-449a/VEGFA axis. First, we detected mRNA and protein levels of VEGFA in HUVECs. As expected, overexpressing circ-100290 in HUVECs upregulated mRNA and protein levels of VEGFA (Fig. 6E and F). Moreover, expression of p-ERK1/2 was also upregulated (Fig. 6F). As such, we examined whether overexpressing miR-449a could rescue the increased expression of VEGFA in hAMSC-CM-treated HUVECs caused by upregulated circ-100290. As expected, results of qPCR and western blot revealed markedly decreased mRNA and protein expression of VEGFA in the oe-circ + miR-449a mimic group compared with the oe-circ + miR-449a mimic-NC group (Fig. 6G and H). Furthermore, the oe-NC + miR-449a mimic group showed the lowest expression of VEGFA (Fig. 6G and H). Notably, expression of p-ERK1/2 exhibited the same trend (Fig. 6H).

Consequently, we wondered whether inhibition of eNOS and VEGFA could regulate the pro-angiogenic role of hAMSC-CM. As such, we next performed a series of assays combining L-NAME and AMG706 (a VEGFR inhibitor) to test this possibility. As expected, migration and tube formation were markedly weakened in the AMG706-treated group compared with the control group (Fig. 6I-K). Moreover, the L-NAME + AMG706 group exhibited the most weakened migration and tube formation (Fig. 6I-K). Thus, both eNOS and VEGFA appear to be potentially important targets of miR-449a in the circ-100290/miR-449a axis, which functions in the pro-angiogenic process in HUVECs induced by hAMSC-CM.

## Discussion

Emerging evidence has elucidated the involvement of circRNAs in pathological processes. For example, circTCF25 was found to regulate the proliferation, migration, and invasion of bladder carcinoma 23, while another study found that circ-HIAT1 promotes the migration and invasion of clear cell renal cell carcinoma 24. In addition to cancer, circRNAs have been implicated in other types of diseases. For instance, circRNA-CER was discovered to be involved in extracellular matrix degradation during osteoarthritis 25; while another investigation concluded that circHIPK3 promotes vascular dysfunction in diabetic retinopathy 26. Beyond disease, circRNAs have also been found to play roles in development. A recent study showed that circRNA-SNX29 regulates the proliferation and differentiation of myoblasts *in vitro* 27. Notably, with regard to angiogenesis, only a few studies have focused on the role of circRNAs in pathologic angiogenesis 17, 18. Indeed, the potential involvement of circRNAs in physiological and therapeutic angiogenesis remains largely unknown. Here, we reported that: 1) circ-100290 was markedly upregulated and involved in the pro-angiogenic induction of HUVECs by hAMSC-CM; 2) circ-100290 may function by binding miR-449a in a pro-angiogenic capacity of hAMSC-CM on HUVECs; and 3) both eNOS and VEGFA are important targets of miR-449a for the pro-angiogenesis effect of hAMSC-CM on HUVECs.

CircRNAs functioning through binding miRNAs was first proposed by Salmena et al. in 2011 28. Since the publication of two subsequent reports focusing on the potential function of circRNAs in 2013 29, 30, study of circRNAs and similar mechanisms have boomed across various fields. With increased investigation of circRNAs, several different patterns of circRNAs functioned through binding miRNAs have been identified with regard to the molecular mechanism of circRNAs. First, most studies revealed a circRNA/miR/mRNA axis underpinning the molecular mechanism of the cellular phenomenon. For example, circPIP5K1A was reported to regulate the progression of non-small cell lung cancer through miR-600/Hif-1α 31. Second, some investigators indicated a circRNA/miR1 +miR2/mRNA axis during their research. For instance, circSLC8A1 was reportedly involved in the progression of bladder cancer by binding miR-130b and miR-494; surprisingly, these two miRNAs cooperatively target PTEN 32. Third, a few researchers discovered a circRNA/miR1/mRNA1 axis and circRNA/miR2/mRNA2 axis that functioned synergistically, such as Circ-SHKBP1, which was found to affect glioma-exposed endothelial cells via miR-544a/FOXP1 and miR-379/FOXP2 axes 18. Notably, very few studies have investigated the fourth potential manner: a circRNA/miR/mRNA1 + mRNA2 axis. Until recently, circular rna‑0054633 was reported to regulate vascular endothelial cells through miR‑218/roundabout 1 and miR‑218/heme oxygenase‑1 axes 17. As miRNAs can target multiple genes in the molecular network of cells, it is possible that these multiple genes might cooperatively contribute to one cellular phenotype. We focused on miR-449a because it not only is predicted to bind circ-100290, also it binds to VEGFA and eNOS 33, 34 which importantly functioned in angiogenesis. In our present study, circ-100290 was found to promote the angiogenesis of HUVECs induced by hAMSC-CM by affecting the mRNA levels of VEGFA and eNOS. Here we report the potential existence of a circ-100290/miR-449a/eNOS + VEGFA axis that functions during the enhancement of angiogenesis in HUVECs induced by hAMSC-CM.

Angiogenesis is an important part of bone regeneration. During angiogenesis, miRNAs play an irreplaceable role. Poliseno et al. first focused on the potential function of miRNAs in angiogenesis of HUVECs in 2006 35. As the investigation of miRNAs involved in angiogenesis deepened, approximately 33 miRNAs have been revealed to have function during angiogenesis in some way 36; miR-449a was not included among them. However, a few studies reported that miR-449a can regulate the migration and invasion of cancer cells 37, 38. Indeed, recent research revealed that miR-449a plays an important role during the endothelial-to-mesenchymal transition of endothelial cells 39. Considering our predicted targets (eNOS and VEGFA) using bioinformatics, we examined the potential involvement of miR-449a in angiogenesis of HUVECs. As our results showed, miR-449a affected both the migration and tube formation of HUVECs treated with hAMSC-CM by targeting eNOS and VEGFA. The role of eNOS in endothelial cells during angiogenesis has attracted significant attention. NO is synthesized from L-arginine by eNOS in endothelial cells, whereby it stimulates soluble guanylyl cyclase to result in increased cyclic guanosine monophosphate, thus activating related downstream signaling pathways to regulate angiogenesis 40, 22. Although circRNA-related regulation of eNOS remains largely unknown, our study revealed the potential involvement of a circ-100290/miR-449a/eNOS pathway in the pro-angiogenesis effect induced by hAMSC-CM on HUVECs.

Promotion of angiogenesis is a new strategy for therapeutic bone regeneration. Indeed, enhancement of therapeutic angiogenesis by stem cells has been revealed as a potential solution 41. Previous studies suggest that MSCs secrete multiple types of relevant growth factors and cytokines, such as VEGF, insulin-like growth factor 1, and hepatocyte growth factor, to promote angiogenesis during bone regeneration in a paracrine manner 42. Similarly, our previous data showed that hAMSCs significantly enhanced angiogenesis of HUVECs potentially through paracrine secretion of matrix metalloproteinases 43. As such, we examined the involvement of circRNAs in this pro-angiogenic process. Our study showed that circ-100290, which was markedly upregulated in HUVECs after induction by hAMSC-CM, functioned by binding miR-449a to act as a pro-angiogenic factor. However, the molecular mechanism underlying enhanced circ-100290 transcription in HUVECs induced by hAMSC-CM remains unknown. It is possible that some related growth factors and cytokines might trigger a signal in HUVECs that result in enhanced circ-100290 transcription. To elucidate the potential upstream molecular mechanism of circ-100290, further investigation is required.

In summary, our study indicated the involvement of circ-100290 in enhanced migration and tube formation of HUVECs induced by hAMSC-CM through binding miR-449a and, thus, upregulated eNOS and VEGFA. Our findings provide insight into the potential molecular mechanism by which hAMSCs induce angiogenesis.

## Supplementary Material

Supplementary figures and tables.

## Figures and Tables

**Figure 7:**
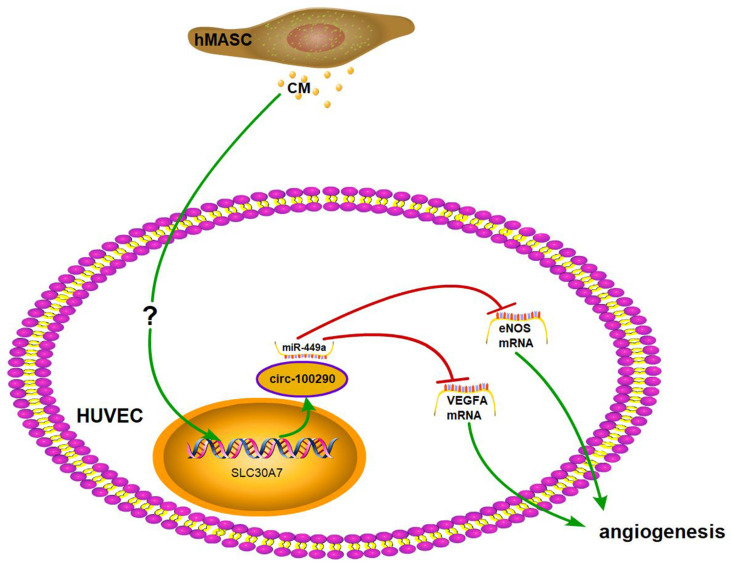
Circ-100290 regulated hAMSC-CM-induced pro-angiogenisis via miR-449a/eNOS and miR-449a/VEGFA axes.

## Abbreviations

circ-100290: circular RNA 100290
CM: conditioned medium
hAMSCs: human amnion-derived mesenchymal stem cells
HUVECs: human umbilical vein endothelial cells
eNOS: endothelial nitric oxide synthase
miRNA or miR: microRNA
MSCs: mesenchymal stem cells
VEGF: vascular endothelial growth factor
