# Supplementary material for: Circ-100290 Positively Regulates Angiogenesis Induced by Conditioned Medium of Human Amnion-Derived Mesenchymal Stem Cells Through miR-449a/eNOS and miR-449a/VEGFA Axes
Source: Int J Biol Sci. 2020 May 18;16(12):2131–44. doi: 10.7150/ijbs.39895 (PMC7294943; doi:10.7150/ijbs.39895)
Supplement: Supplementary file 1 — Supplementary figures and tables. [file ijbsv16p2131s1.pdf]

Table S1. Sequences of qPCR primers

**Primers for qPCR (5’-3’)**	
circ 100290- F	ACTTTCATTCTCATGCTTAGGCT
circ 100290- R	AGTCCAGCCAAAATGGCAGT
SLC30A7-F	TTGCCATAGCCATGAAGTGA
SLC30A7-R	GTCTGCTGGGTCCTGTTGTT
eNOS- F	AGGAACCTGTGTGACCCTCA
eNOS- R	CGAGGTGGTCCGGGTATCC
VEGFA- F	AGGGCAGAATCATCACGAAGT
VEGFA- R	AGGGTCTCGATTGGATGGCA
GAPDH- F	GACTCATGACCACAGTCCATGC
GAPDH- R	AGAGGCAGGGATGATGTTCTG
U6- F	GCTCGCTTCGGCAGCACATAT
U6- R	ATATGGAACGCTTCACGAATTTGC
U6-RT	GTCGTATCCAGTGCAGGGTCCGAGGTATTCGCACTGGATACGACAAAATA
miR-449a RT	GTCGTATCCAGTGCAGGGTCCGAGGTATTCGCACTGGATACGACACCAGC
miR-449a F	CGGCGGTTGGCAGTGTATTGTTA
miR-449a R	CCAGTGCAGGGTCCGAGGTAT
**miR-mimic and miR-inhibitor (5’-3’)**	
miR-449a mimic	UGGCAGUGUAUUGUUAGCUGGU
miR-mimic NC	ACGUGACACGUUCGGAGAAUU
miR-449a inhibitor	ACCAGCTAACAATACACTGCCA
mir-inhibitor NC	CAGUACUUUUGUGUAGUACAA
**siRNA and oe-circ (5’-3’)**	
si-1	ATTCTCATGCTTAGGCTTGAT
si-2	TTCATTCTCATGCTTAGGCTT
si-3	TCTCATGCTTAGGCTTGATTT
si-NC	CACAGTCAAAAGATGTTGGTT
**vectors for Dual-Luciferase reporter assay (5’-3’)**	
circ-100290-MUT	43-49 CACTGCC >ACAGTAA
VEGFA-MUT	859-865 CACTGCC>ACAGTAA
eNOS-MUT	250-256 ACTGCC>CAGTAA
